# Supplementary material for: Molecular recognition of an acyl-peptide hormone and activation of ghrelin receptor
Source: Nat Commun. 2021 Aug 20;12:5064. doi: 10.1038/s41467-021-25364-2 (PMC8379176; doi:10.1038/s41467-021-25364-2)
Supplement: Supplementary file 1 — Supplementary Information [file 41467_2021_25364_MOESM1_ESM.pdf]

Supplementary Information

Molecular recognition of an acyl-peptide hormone and activation of ghrelin receptor

Supplementary Figure 1. Ghrelin receptor construct and $G\alpha_q$ chimera/mutant constructs used in this study.

Supplementary Figure 2. The comparison of wild-type or engineered ghrelin receptor response for ghrelin.

Supplementary Figure 3. Ghrelin receptor–G_q complex purification and cryo-EM data processing.

Supplementary Figure 4. Overall resolution analysis of electron density of transmembrane helices, α5-helix of G_q, ghrelin and GHRP-6.

Supplementary Figure 5. Sequence alignment of the ghrelin receptor family.

Supplementary Figure 6. Saturation experiments of ghrelin-A2 binding to the WT and mutant ghrelin receptor.

Supplementary Figure 7. Ghrelin/GHRP-6 response curves for WT or ghrelin receptor mutants.

Supplementary Figure 8. Structural comparison of G_q-coupled ghrelin receptors with other $G_{q/11}$-coupled GPCRs solved to date.

Supplementary Figure 9. Conformational changes of the extracellular end of TM7 across representative class A GPCRs.

Supplementary Figure 10. Effects of alanine mutations of residues on the basal activity of the ghrelin receptor.

Supplementary Figure 11. Gating strategy of cell surface expression assay.

Supplementary Table 1. Cryo-EM data collection, model refinement, and validation statistics.

Supplementary Table 2. Kd and B_{max} of ghrelin-A2 binding to ghrelin receptor mutants and EC_{50} values of different ligands on ghrelin receptor mutants.

Supplementary Table 3. List of primers sequences for site-direct mutagenesis studies.

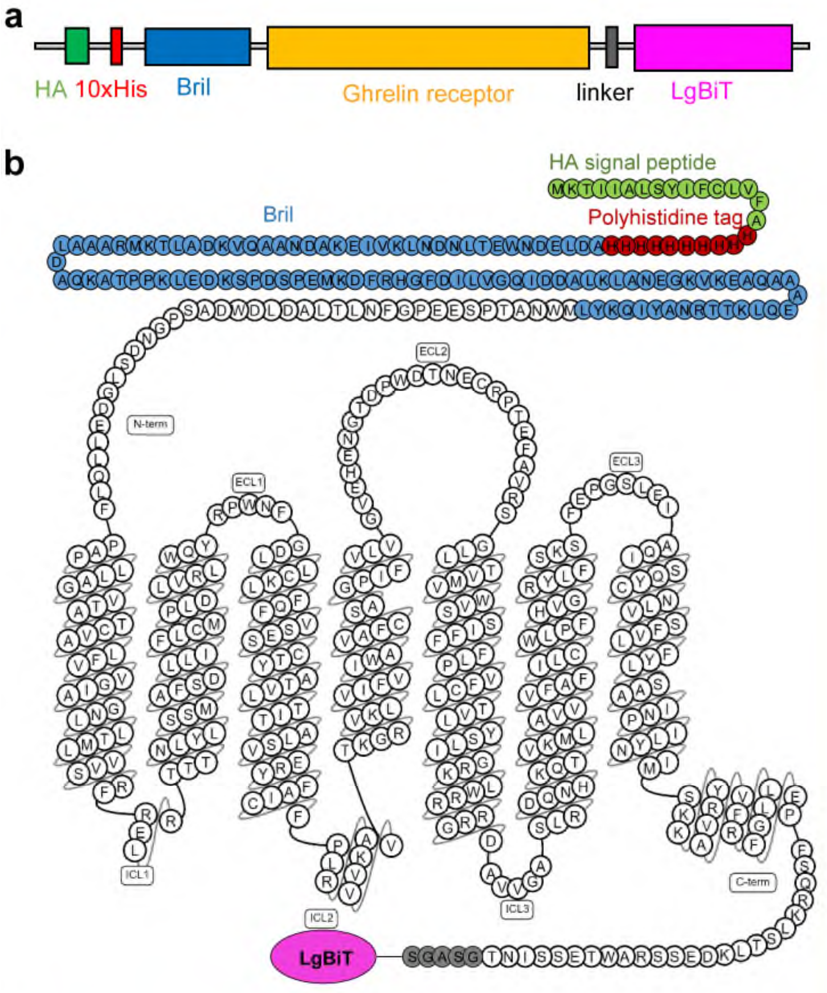

c

MMGCTLSAEDKAAVERSKMIEKQLQKDKQVYRRTLRLLLLGADNSGKSTIVKQMRIYHVN
GYSEEECKQYKAVVYSNTIQSIIAIIRAMGRLKIDFGDSARADDARQLFVLAGAAEEGFMTA
ELAGVIKRLWKDSGVQACFNRSREYQLNDSAAYYLNDLDRIAQPNYIPTQQDVLRTRVKT
SGIFETKFQVDKVNFHMFDVGAQRDERRKWIQCFNDVTAIIFVVDSSDYNRLQEALNDFKS
IWNNRWLRTISVILFLNKQDLLAEKVLAGKSKIEDYFPEFARYTTPEDATPEPGEDPRVTRA
KYFIRKEFVDISTASGDGRHICYPHFTCSVDTENARRIFNDCKDIILQMNLREYNLV

Supplementary Figure 1 | Ghrelin receptor construct and Gα_q chimera/mutant constructs used in this study. a, **b**, Schematic representation (**a**) and snake model (**b**) of the ghrelin receptor construct. **c**, Sequence of engineered Gα_q, the skeleton is based on miniGα_s (for Nb35 binding), which is shown in black. N-terminus in red is replaced by Gα_{i1} (for scFv16 binding). Two dominant-negative mutations are shown in cyan, WT Gα_q is colored in magenta.

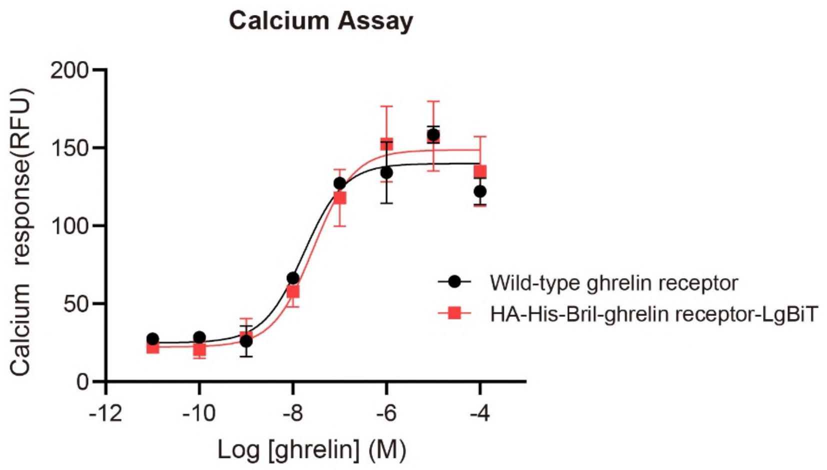

Groups	pEC_{50}
Wild-type ghrelin receptor	7.77
HA-His-Bril-ghrelin receptor-LgBiT	7.56

Supplementary Figure 2 | The comparison of wild-type or engineered ghrelin receptor response for ghrelin. The engineered HA-His-Bril-ghrelin receptor-LgBiT was the mutant used in structural studies of ghrelin-G_q complexes. Data represent mean ± S.E.M. of three biological replicates (n=3).

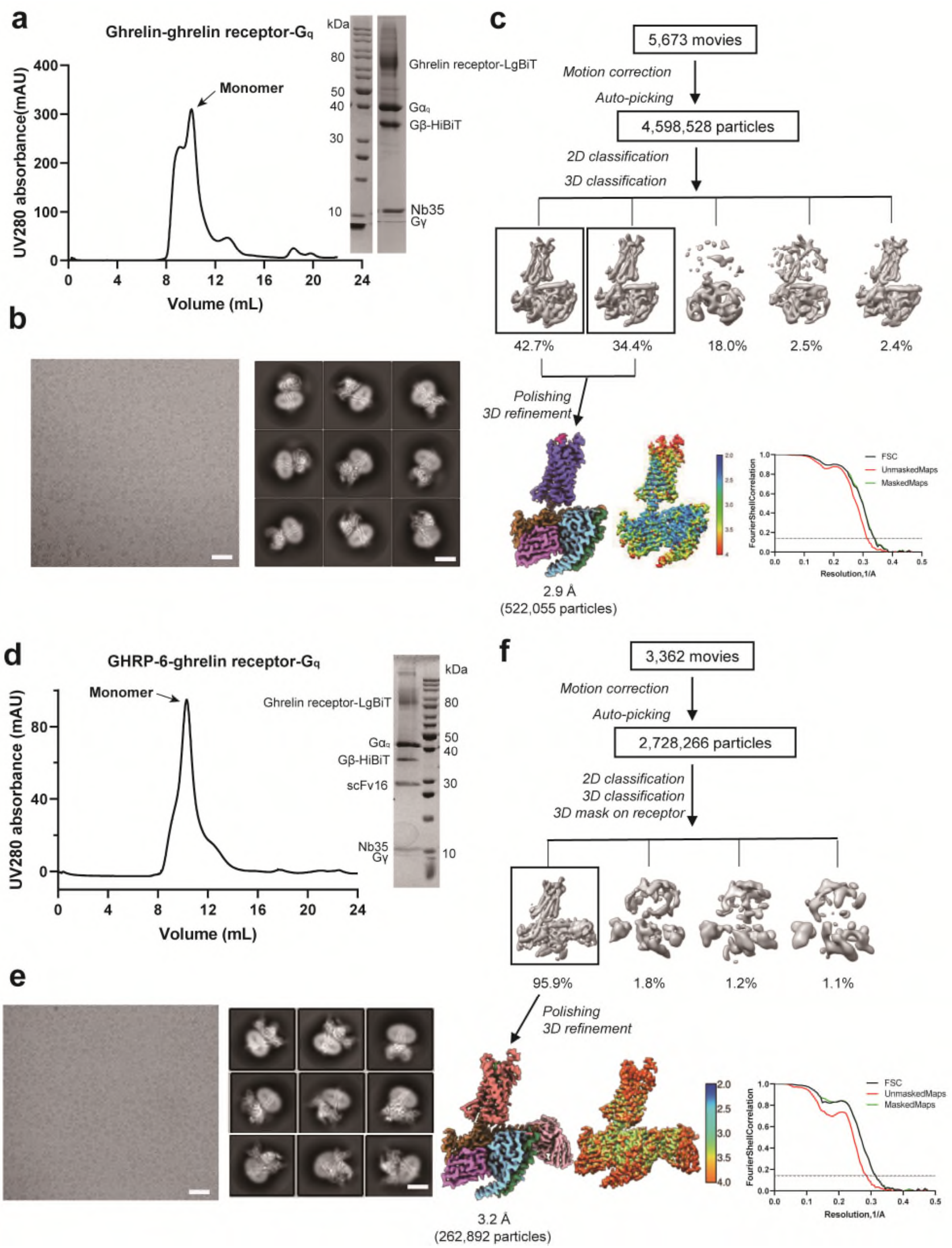

Supplementary Figure 3 | Ghrelin receptor–G_q complex purification and cryo-EM data processing. a, Representative elution profile of His-purified ghrelin–ghrelin receptor–LgBiT–G_q complex and SDS–PAGE of the size-exclusion chromatography peak. **b**, Cryo-EM micrographs of ghrelin–ghrelin receptor–LgBiT–G_q complex (scale bar: 50 nm) and 2D class averages (scale bar: 5 nm). The data collection was performed once. **c**, Flow chart of the cryo-EM data processing for the ghrelin–ghrelin receptor–LgBiT–G_q complex. **d**, Representative elution profile of His-purified GHRP-6–ghrelin receptor–LgBiT–G_q complex and SDS–PAGE of the size-exclusion chromatography peak. The data collection was performed once. **e**, Cryo-EM micrographs of GHRP-6–ghrelin receptor–LgBiT–G_q complex (scale bar: 50 nm) and 2D class averages (scale bar: 5 nm). **f**, Flow chart of the cryo-EM data processing for the GHRP-6–ghrelin receptor–LgBiT–G_q complex.

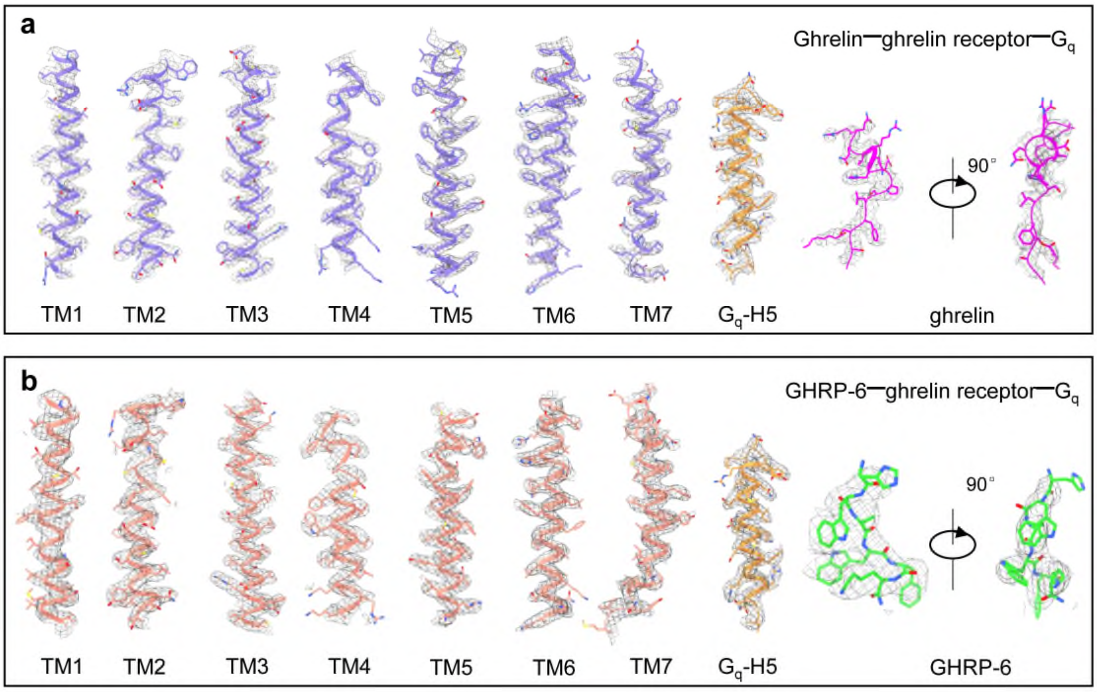

Supplementary Figure 4 | Overall resolution analysis of electron density of transmembrane helices, α5-helix of G_q, ghrelin and GHRP-6. a, EM density and model of all transmembrane helices of ghrelin-bound ghrelin receptor. **b**, GHRP-6-bound ghrelin receptor and α5 helix of $G\alpha_q$ subunit.

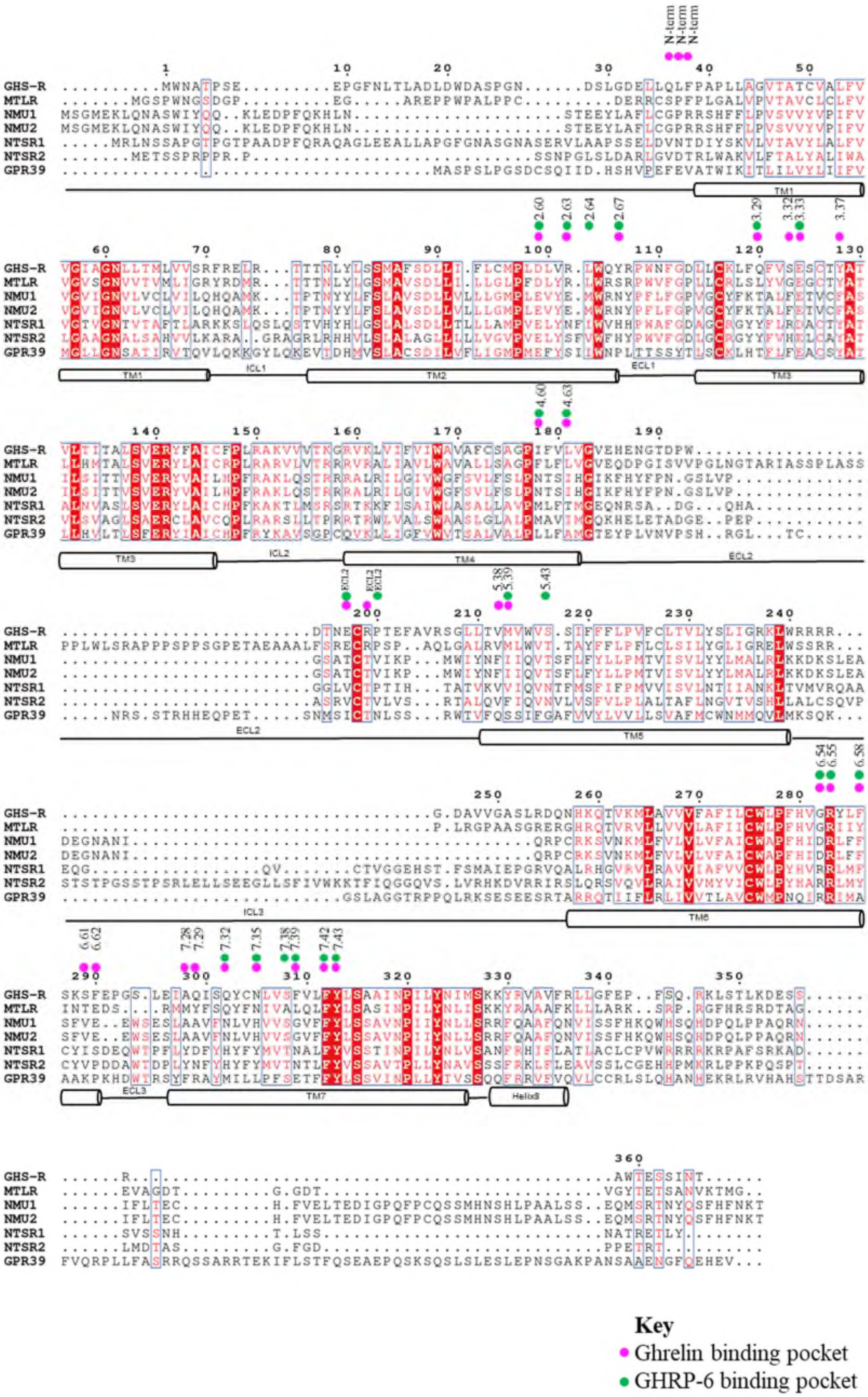

Supplementary Figure 5 | Sequence alignment of the ghrelin receptor family. The sequences shown are those for motilin receptor (MTLR), neuromedin U receptor 1/2 (NMU1/2), neurotensin receptors 1/2 (NTSR1/2) and orphan receptor (GPR39), The sequence alignment was created using Clustalw and ESPript 3.0 servers. α-helices for ghrelin receptor are shown as columns. The binding-pocket residues are shown as magenta (ghrelin) and green (GHRP-6) dots.

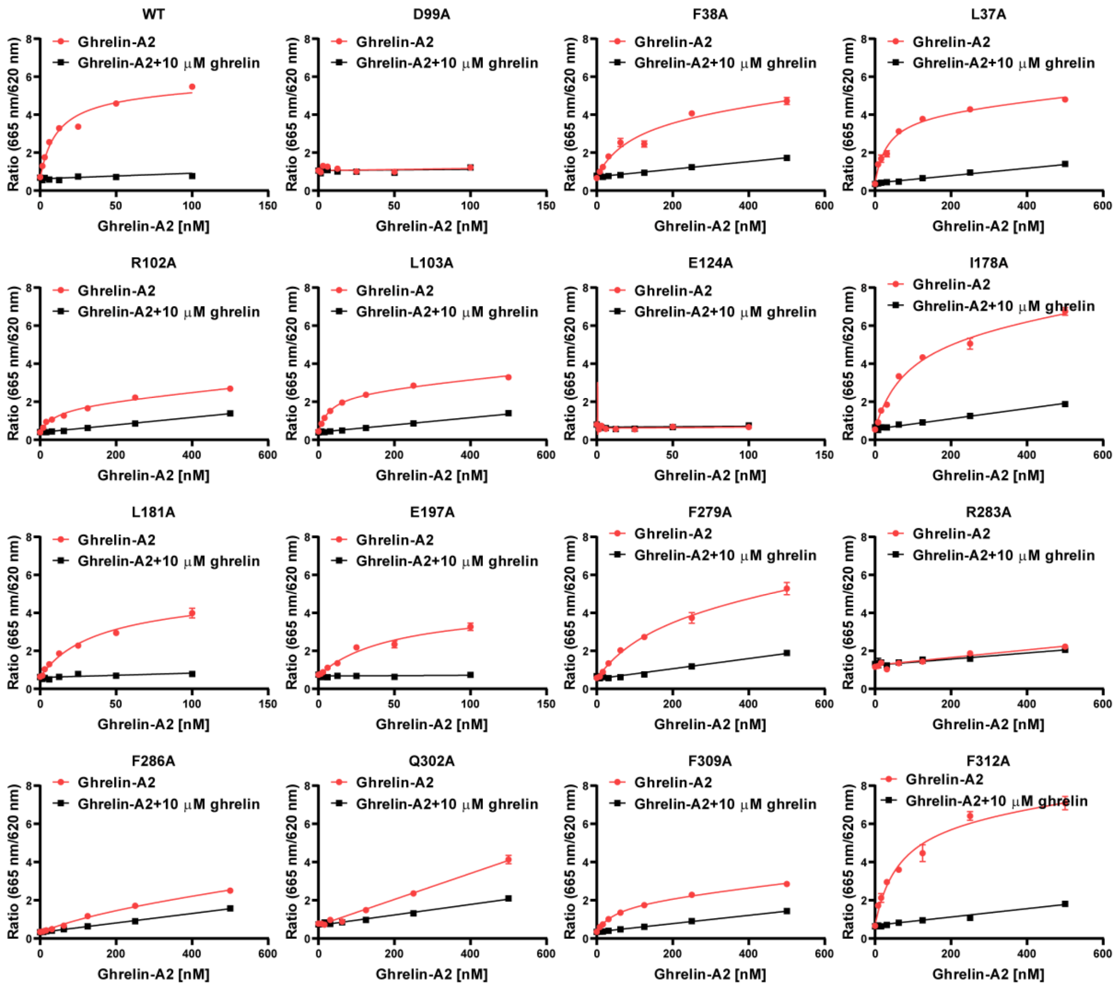

Supplementary Figure 6 | Saturation experiments of ghrelin-A2 binding to the WT and mutant ghrelin receptor. Effects of different mutations within the ligand-binding pocket of ghrelin receptor. Amino acids located in the ghrelin binding pocket were mutated to an alanine residue, saturation binding experiments on HEK293 cells were transfected with different ghrelin receptor mutations. Each point represents mean ± S.E.M. from three or four independent experiments. Saturation experiments for WT, D99A, E124A, L181A, and E197A are performed in quadruplicate (n=4), while experiments for other ghrelin receptor mutants were conducted in triplicate (n=3).

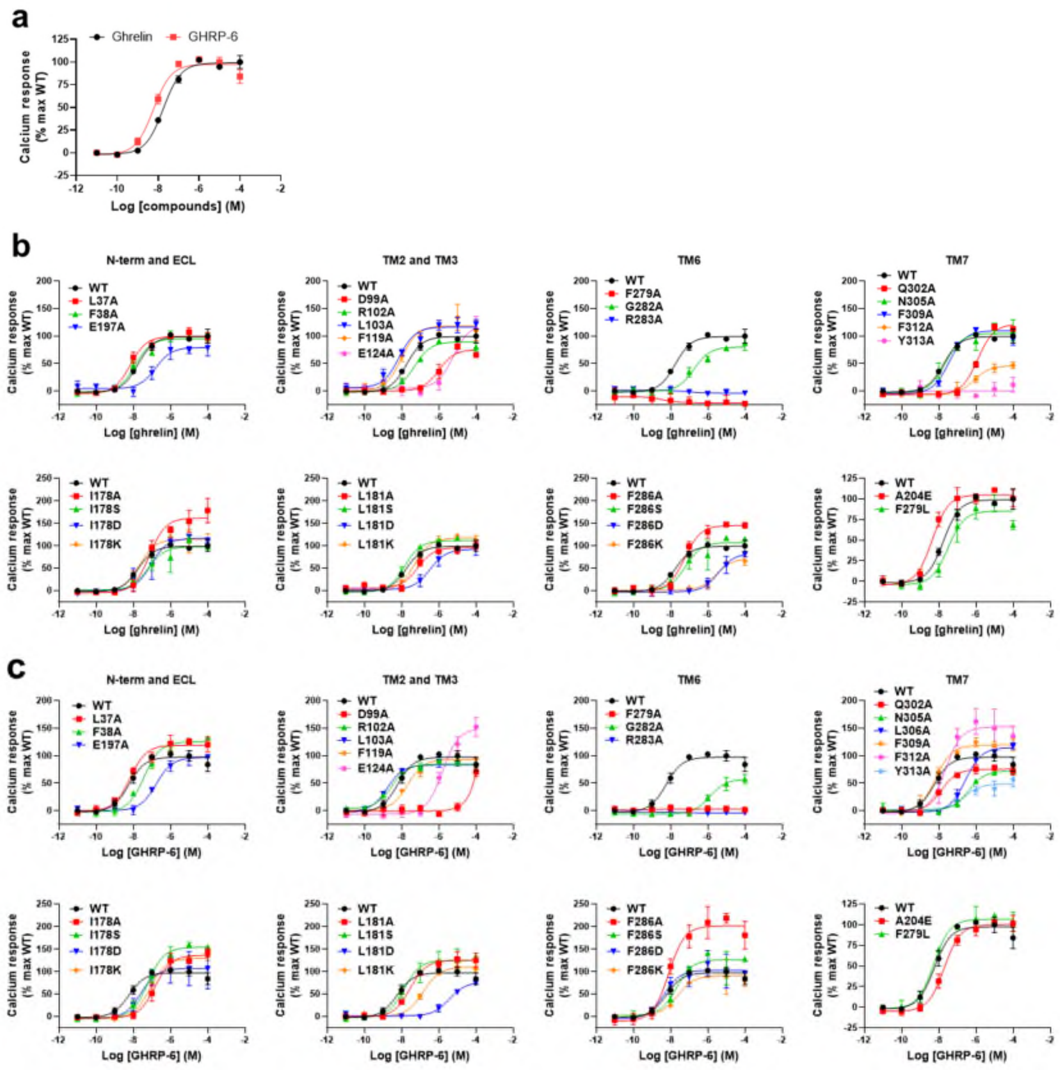

Supplementary Figure 7 | Ghrelin/GHRP-6 response curves for WT or ghrelin receptor mutants. HEK293 cells were transfected with WT or ghrelin receptor mutant constructs. Intracellular calcium signals were monitored after stimulation with ghrelin or GHRP-6. Each point represents mean ± S.E.M. from three independent experiments. **a**, Comparison of the activities of ghrelin or GHRP-6 on WT ghrelin receptor. GHRP-6 displays comparable potency with ghrelin for ghrelin receptor activation. **b**, Effects of ghrelin receptor mutations on ghrelin induced calcium mobilization. c, Effects of ghrelin receptor mutations on GHRP-6 induced calcium mobilization.

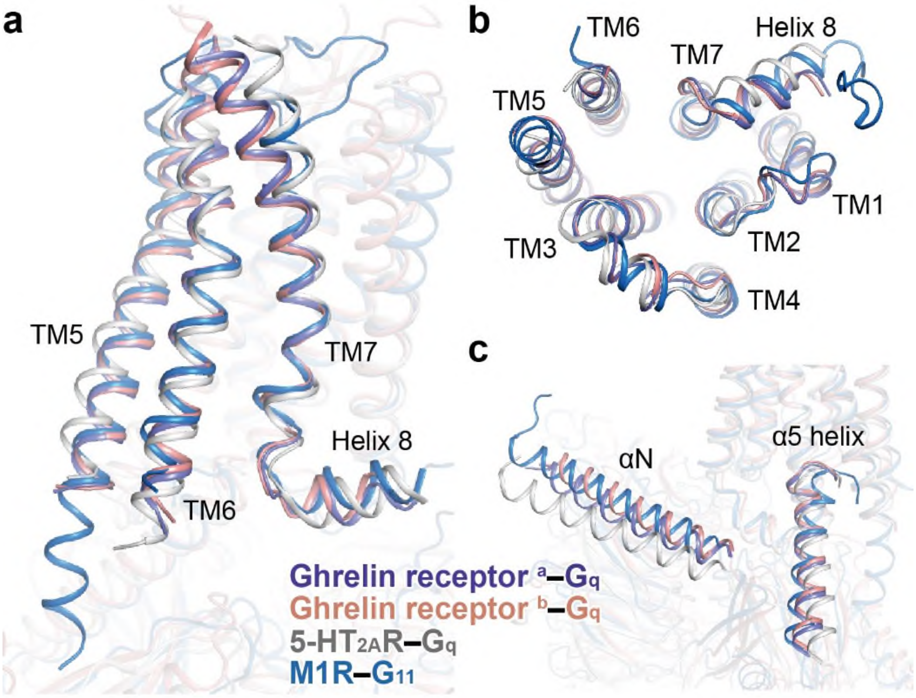

Supplementary Figure 8 | Structural comparison of G_q-coupled ghrelin receptors with other $G_{q/11}$-coupled GPCRs solved to date. a, **b**, Structural superpositions of $G_{q/11}$-coupled receptors. Orthogonal view (**a**), extracellular view (**b**). Structures of TMs 5-7 from receptors are highlighted. **c**, Conformational comparison of αN and α5 helix of $G_{q/11}$ proteins. αN, N-terminus of $G\alpha_q$ subunit. [a], ghrelin-bound ghrelin receptor complex (slate blue). [b], GHRP-6-bound ghrelin receptor complex (salmon). G_q-coupled 5-HT_{2A}R is shown in grey (PDB: 6WHA), and G_{11}-coupled M1R is displayed in marine (PDB: 6OIJ). The Ligands are omitted for clear presentation.

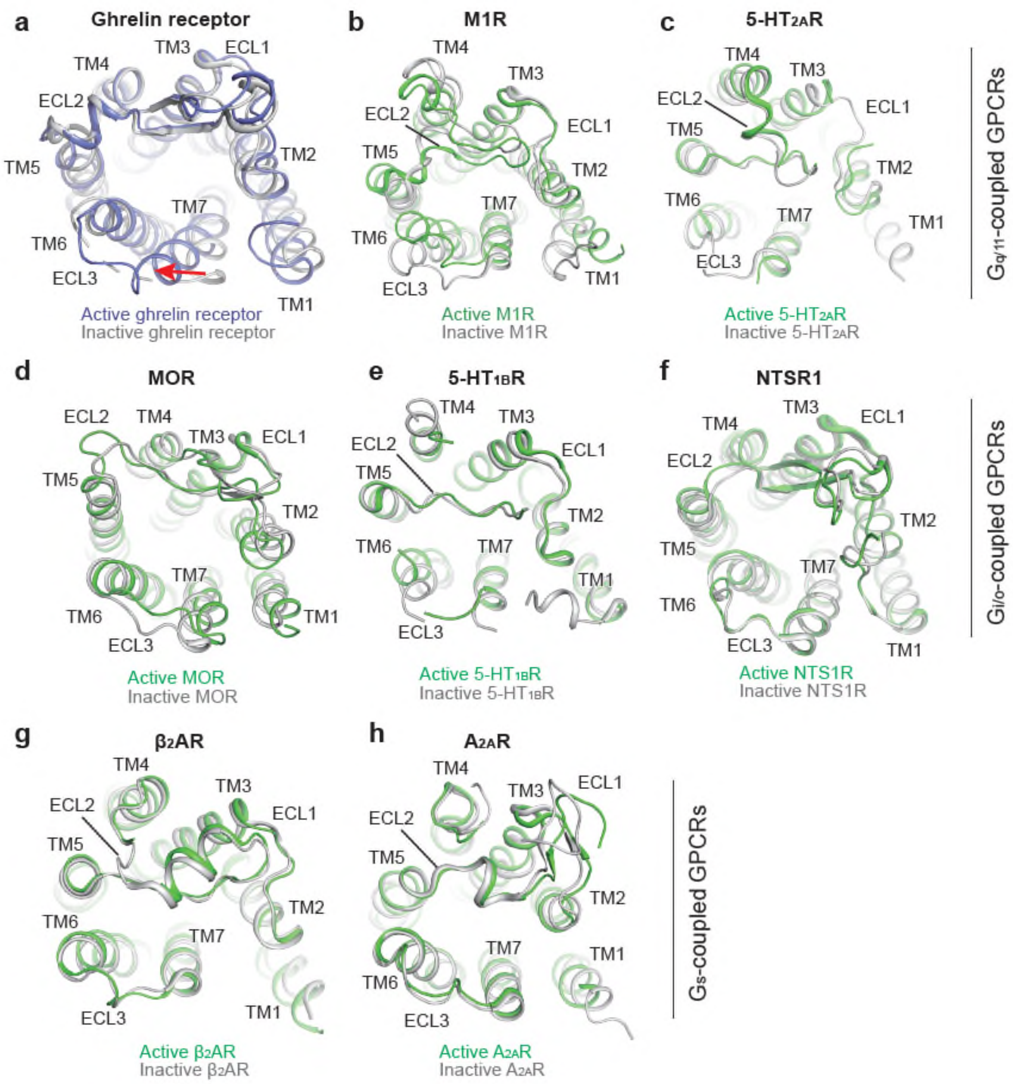

Supplementary Figure 9 | Conformational changes of the extracellular end of TM7 across representative class A GPCRs. Structural superposition of $G_{q/11}$-coupled (**a-c**), $G_{i/o}$-coupled (**d-f**), and G_s-coupled GPCRs (**g, h**) in the extracellular view. **a**, active ghrelin receptor (ghrelin-bound) and antagonist-bound ghrelin receptor; **b**, Active M1R (PDB: 6OIJ) and inactive M1R (PDB: 6WJC); **c**, Active 5-HT_{2A}R (PDB: 6WHA) and inactive 5-HT_{2A}R (PDB: 6A94); **d**, Active MOR (PDB: 6DDE) and inactive MOR (PDB: 4DKL); **e**, Active 5-HT_{1B}R (PDB: 5G79) and inactive 5-HT_{1B}R (PDB: 5V54); **f**, Active NTSR1 (PDB: 4GRV) and inactive NTSR1 (PDB: 4BUO); **g**, Active β_2AR (PDB: 3SN6) and inactive β_2AR (PDB: 3NYA); **h**, Active A_{2A}R (PDB: 5G53) and inactive A_{2A}R (PDB: 3EML). Except for active ghrelin receptor (slate blue), other active receptors are colored in green, and all inactive or antagonist-bound receptors are shown in grey.

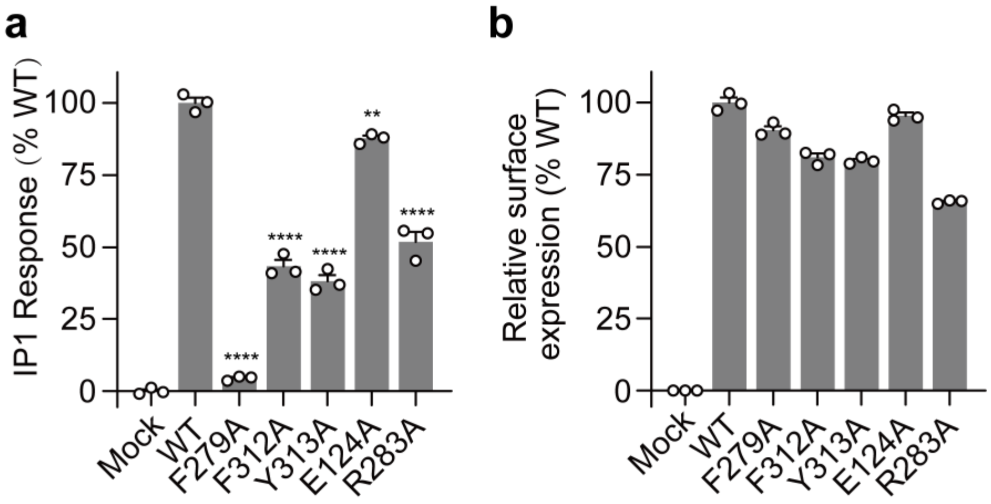

Supplementary Figure 10 | Effects of alanine mutations of residues on the basal activity of the ghrelin receptor. Inositol phosphate accumulation assay was performed to evaluate the basal activity of the ghrelin receptor (**a**). Residues in the "hydrophobic lock" (F279$^{6.51}$, F312$^{7.42}$, and Y313$^{7.43}$) and residues forming a salt bridge (E124$^{3.33}$ and R283$^{6.55}$) were substituted with alanine. The cell surface expression of these mutants was determined by using flow cytometry (**b**). Considering the Y313A mutation decreases the cell surface expression, a 4-fold amount of the Y313A construct relative to other mutants was transfected into HEK-293 cells to achieve a comparable expression level compared with wild-type (WT) receptor. Data are presented as means ± S.E.M. of three independent experiments (n=3) performed in technical triplicate. All Inositol phosphate accumulation signals were analyzed by two-side, one-way ANOVA with Tukey's test (P<0.0001, P<0.0001, P<0.0001, P=0.0047, and P<0.0001 for ghrelin receptor mutants from left to right). $^{**}P$<0.01, $^{****}P$<0.0001 *vs.* WT receptor.

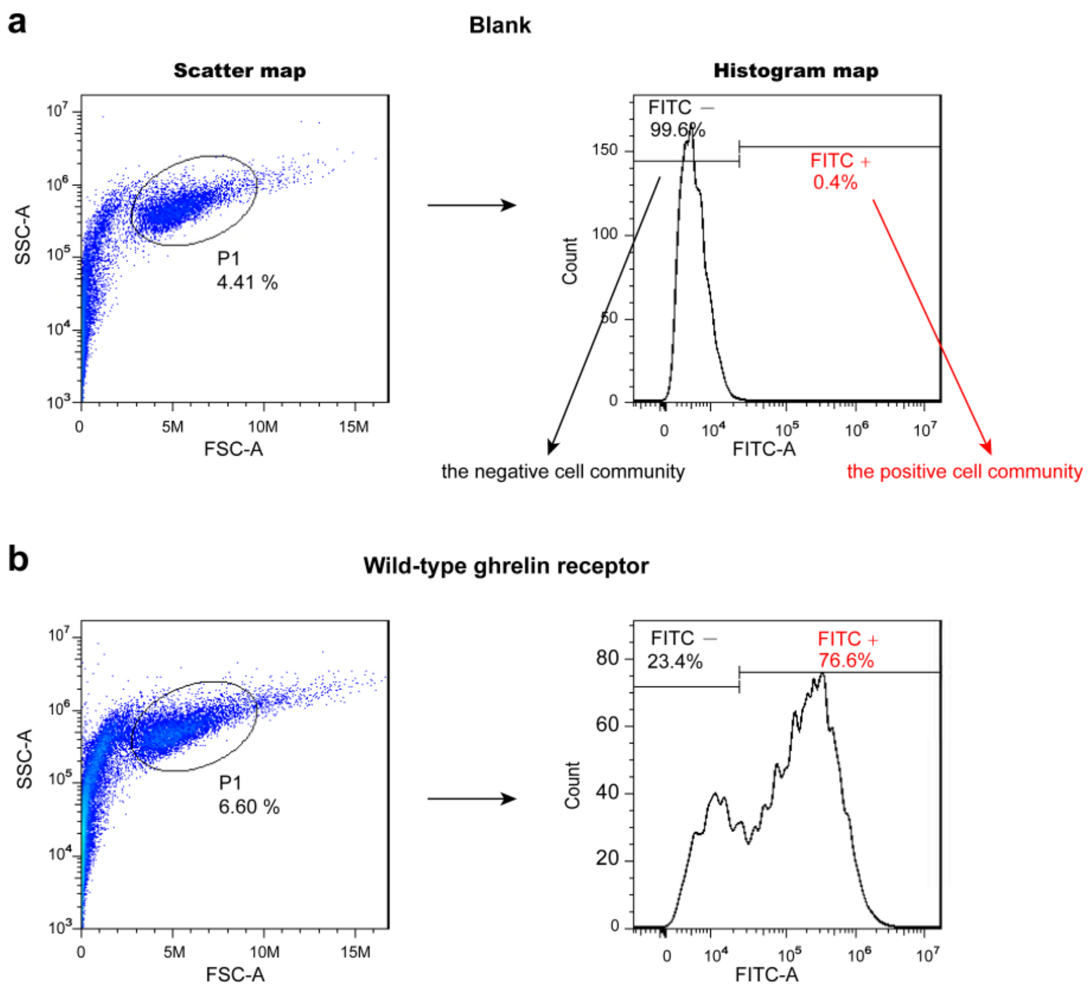

Supplementary Figure 11 | Gating strategy of cell surface expression assay. Circle a gate P1 in the scatter map (black circle). The cells shown in the histogram map are all the cells in the gate P1 in the scatter map. Fluorescence signal intensity (FITC) is presented by scatter map. With the Blank sample as the reference value of background fluorescence signal (**a**), the fluorescence signal histogram map is divided into two regions. The left region represents the negative cell community ($FITC^-$), while the right region represents the positive cell community ($FITC^+$). The expression level of cell surface wild-type (WT) ghrelin receptor (**b**) can be calculated as follows: $(M(FITC^+)\text{-}M(FITC^-)) \times (FITC^+\%\ Parent)$. M, mean fluorescence intensity. The expression level of the ghrelin receptor mutant is calculated similarly to WT receptor and then is normalized with the WT to calculate the relative expression value.

Supplementary Table 1 | Cryo-EM data collection, model refinement, and validation statistics

Ghrelin receptor-G_q	Ghrelin bound (EMD-31500) (PDB 7F9Y)	GHRP-6 bound (EMD-31501) (PDB 7F9Z)
Data Collection and Processing		
Magnification	81,000	81,000
Voltage (kV)	300	300
Electron exposure (e^-/Å^2)	80	80
Defocus range (μm)	-0.5 to -3.0	-0.5 to -3.0
Pixel size (Å)	1.045	1.045
Symmetry imposed	C1	C1
Initial particle projections (no.)	4,598,528	2,728,266
Final particle projections (no.)	522,055	262,892
Map resolution (Å)	2.9	3.2
FSC threshold	0.143	0.143
Map resolution range (Å)	2.5-5.0	2.5-5.0
Refinement		
Initial model used (PDB accession number)	6OSA	6OSA
Model resolution (Å)	3.3	3.7
FSC threshold	0.5	0.5
Model resolution range (Å)	50-2.9	50-3.2
Map sharpening B factor (Å^2)		
Model Composition		
Non-hydrogen atoms	8264	9987
Protein residues	1052	1265
Ligand	1	1
Lipids	2	0
B factors (Å^2)		
Protein	102.4	119.83
Ligand	89.5	92.67
RMSD		
Bound lengths (Å)	0.002	0.002
Bound angles (°)	0.480	0.572
Validation		
MolProbity score	1.25	1.46
Clashscore	4.7	6.3
Rotamer outliers (%)	0	0
Ramachandran Plot		
Favored (%)	98.84	97.43
Allowed (%)	1.16	2.57
Disallowed (%)	0	0

Supplementary Table 2 | *Kd* and B_{max} of ghrelin-A2 binding to ghrelin receptor mutants and EC_{50} values of different ligands on ghrelin receptor mutants.

	Ligand-binding assays			Calcium assay				Cell-surface expression
	ghrelin-A2			ghrelin		GHRP-6		
	Kd (nM)	fold of change	B_{max} (% max WT)	EC_{50} (nM)	fold of change	EC_{50} (nM)	fold of change	Relative to WT
WT	10.84±0.19	1	100	18.0±1.2	1	5.97±0.6	1	100±1.9
L37$^{N\text{-term}}$A	32.9±0.44	3	80.8	7.33±1.43	0.4	6.94±1.16	1.2	98.43±0.8
F38$^{N\text{-term}}$A	99.27±16.5	9.2	77.1	12.6±2.32	0.7	39.9±6.92	6.7	93.64±0.5
D99$^{2.60}$A	UD	+++	UD	1121.3±335.1	62.3	UD	+++	95.09±1.9
R102$^{2.63}$A	41.9±2.33	3.9	30.8	47.6±6.78	2.6	2.86±0.33	0.5	96.7±2.5
L103$^{2.64}$A	30.9±0.23	2.9	45.7	7.02±2.28	0.4	1.62±0.11	0.3	99.11±2.3
E124$^{3.33}$A	UD	+++	UD	6547±2390	363.7	1646±426	275.7	93.73±1.3
I178$^{4.60}$A	84.30±1.87	7.8	117.5	76.1±6.9	4.2	154.7±23.5	25.8	94.6±1.9
I178$^{4.60}$S	NT	NT	NT	67.2±11.1	3.7	63±7.2	10.6	100.18±1
I178$^{4.60}$D	NT	NT	NT	96.4±21.3	5.4	25.4±1.8	4.3	88.05±0.8
I178$^{4.60}$K	NT	NT	NT	22.5±4	1.3	79.2±5.7	13.3	93.22±2.1
L181$^{4.63}$A	37.75±2.5	3.5	91	75.2±7	4.2	29.9±3.8	5	99.53±3.5
L181$^{4.63}$S	NT	NT	NT	14.9±1.5	0.8	16.9±5.8	2.8	95.13±1.2
L181$^{4.63}$D	NT	NT	NT	368.5±59.9	20.4	3854±512.6	64.5	97.24±1
L181$^{4.63}$K	NT	NT	NT	50.1±3.5	2.8	151.1±11.8	25.3	89.91±1.1
E197^{ECL2}A	50.88±0.25	4.7	81.4	161.7±33.5	8.9	173±9.9	29	103.8±1.7
F279$^{6.51}$A	180.1±23.7	16.7	98.1	UD	+++	UD	+++	101.27±1.9
G282$^{6.54}$A	NT	NT	NT	225±93.0	12.5	1387.6±352.4	232.3	90.13±3.5
R283$^{6.55}$A	UD	+++	UD	UD	+++	UD	+++	63.24±1.8
F286$^{6.58}$A	275±27.5	25.5	32.7	57.9±2.8	3.2	8.1±0.9	1.4	91.26±0.9
F286$^{6.58}$S	NT	NT	NT	75.2±21.1	4.2	21.2±2.7	3.6	99.6±0.6
F286$^{6.58}$D	NT	NT	NT	4661±1415	258.9	4.6±0.5	0.8	95.36±1
F286$^{6.58}$K	NT	NT	NT	2966±46.1	164.8	24.6±3.3	4.1	101.09±1
S289$^{6.61}$A	NT	NT	NT	19.9±6.41	1.1	1.89±0.44	0.3	99.84±1.5
Q302$^{7.32}$A	>500	+++	UD	1267±48.7	70.3	13.7±0.5	2.3	92.67±3
N305$^{7.35}$A	NT	NT	NT	24.6±7.43	1.4	420±50.5	70.4	85.3±1.2
F309$^{7.39}$A	52.5±0.85	4.9	34.4	36.5±4.25	2	7.01±0.82	1.2	79.97±1.5
F312$^{7.42}$A	64.47±8.60	6	128.8	794±148	43.8	23±2.9	3.9	82±2.3
Y313$^{7.43}$A	NT	NT	NT	UD	+++	326±160	54.6	25.14±0.8
A204^{ECL2}E	NT	NT	NT	4.67±0.59	0.3	21.1±3.60	3.5	78.78±1.1
F279$^{6.51}$L	NT	NT	NT	41.4±16.3	2.3	5.51±0.39	0.9	99.52±1

UD, Undetectable; +++, Exceedingly high fold of change due to the undetectable or very low agonist activity; NT, Not tested.

Supplementary Table 3. List of primers sequences for site-direct mutagenesis studies.

Oligonucleotides primer	Forward	Reverse
L37$^{N\text{-}term}$A	CTCCAGGCGTTCCCAGCTCCTCTGCTGGC	CTCCAGGCGTTCCCAGCTCCTCTGCTGGC
F38$^{N\text{-}term}$A	CAGCTGGCCCCAGCTCCTCTGCTGGCTGG	CAGCTGGCCCCAGCTCCTCTGCTGGCTGG
D99$^{2.60}$A	CCTCTGGCCCTGGTCCGTCTGTGGCAGTAC	CCTCTGGCCCTGGTCCGTCTGTGGCAGTAC
R102$^{2.63}$A	CTGGTCGCTCTGTGGCAGTACCGCCCTTGG	CTGGTCGCTCTGTGGCAGTACCGCCCTTGG
L103$^{2.64}$A	GTCCGTGCGTGGCAGTACCGCCCTTGGAAC	GTCCGTGCGTGGCAGTACCGCCCTTGGAAC
E124$^{3.33}$A	GTGTCCGCGAGCTGCACCTACGCCACCGTG	GTGTCCGCGAGCTGCACCTACGCCACCGTG
I178$^{4.60}$A	GGACCAGCCTTCGTTCTCGTGGGAGTTGAA	GGACCAGCCTTCGTTCTCGTGGGAGTTGAA
I178$^{4.60}$S	GGACCATCCTTCGTTCTCGTGGGAGTTGAA	GGACCATCCTTCGTTCTCGTGGGAGTTGAA
I178$^{4.60}$D	GGACCAGACTTCGTTCTCGTGGGAGTTGAA	GGACCAGACTTCGTTCTCGTGGGAGTTGAA
I178$^{4.60}$K	GGACCAGAGTTCGTTCTCGTGGGAGTTGAA	GGACCAGAGTTCGTTCTCGTGGGAGTTGAA
L181$^{4.63}$A	TTCGTTGCCGTGGGAGTTGAACACGAAAAC	TTCGTTGCCGTGGGAGTTGAACACGAAAAC
L181$^{4.63}$S	TTCGTTTCCGTGGGAGTTGAACACGAAAAC	TTCGTTTCCGTGGGAGTTGAACACGAAAAC
L181$^{4.63}$D	TTCGTTGACGTGGGAGTTGAACACGAAAAC	TTCGTTGACGTGGGAGTTGAACACGAAAAC
L181$^{4.63}$K	TTCGTTGAGGTGGGAGTTGAACACGAAAAC	TTCGTTGAGGTGGGAGTTGAACACGAAAAC
E197^{ECL2}A	ACTAACGCATGTAGACCTACTGAATTCGCT	ACTAACGCATGTAGACCTACTGAATTCGCT
F279$^{6.51}$A	CTGCCTGCCCACGTGGGTCGCTACCTGTTC	CTGCCTGCCCACGTGGGTCGCTACCTGTTC
G282$^{6.54}$A	CACGTGGCTCGCTACCTGTTCAGCAAGTCC	CACGTGGCTCGCTACCTGTTCAGCAAGTCC
R283$^{6.55}$A	GTGGGTGCCTACCTGTTCAGCAAGTCCTTC	GTGGGTGCCTACCTGTTCAGCAAGTCCTTC
F286$^{6.58}$A	TACCTGGCCAGCAAGTCCTTCGAACCAGGC	TACCTGGCCAGCAAGTCCTTCGAACCAGGC
F286$^{6.58}$S	TACCTGTCCAGCAAGTCCTTCGAACCAGGC	TACCTGTCCAGCAAGTCCTTCGAACCAGGC
F286$^{6.58}$D	TACCTGGACAGCAAGTCCTTCGAACCAGGC	TACCTGGACAGCAAGTCCTTCGAACCAGGC
F286$^{6.58}$K	TACCTGGAAAGCAAGTCCTTCGAACCAGGC	TACCTGGAAAGCAAGTCCTTCGAACCAGGC
S289$^{6.61}$A	AGCAAGGCCTTCGAACCAGGCTCTCTCGA	AGCAAGGCCTTCGAACCAGGCTCTCTCGA
Q302$^{7.32}$A	ATCTCCGCGTACTGTAACCTGGTGAGCTTC	ATCTCCGCGTACTGTAACCTGGTGAGCTTC
N305$^{7.35}$A	TACTGTGCCCTGGTGAGCTTCGTGCTGTTC	TACTGTGCCCTGGTGAGCTTCGTGCTGTTC
F309$^{7.39}$A	GTGAGCGCCGTGCTGTTCTACCTGTCTGCT	GTGAGCGCCGTGCTGTTCTACCTGTCTGCT
F312$^{7.42}$A	GTGCTGGCCTACCTGTCTGCTGCCATCAAC	GTGCTGGCCTACCTGTCTGCTGCCATCAAC
Y313$^{7.43}$A	CTGTTCGCCCTGTCTGCTGCCATCAACCCT	CTGTTCGCCCTGTCTGCTGCCATCAACCCT
A204^{ECL2}E	GAATTCGAGGTGCGTTCTGGATTGCTGAC	GAATTCGAGGTGCGTTCTGGATTGCTGAC
F279$^{6.51}$L	CTGCCTTTACACGTGGGTCGCTACCTGTT	CTGCCTTTACACGTGGGTCGCTACCTGTT
